# Interlayer orientation-dependent light absorption and emission in monolayer semiconductor stacks

**DOI:** 10.1038/ncomms8372

**Published:** 2015-06-23

**Authors:** Hoseok Heo, Ji Ho Sung, Soonyoung Cha, Bo-Gyu Jang, Joo-Youn Kim, Gangtae Jin, Donghun Lee, Ji-Hoon Ahn, Myoung-Jae Lee, Ji Hoon Shim, Hyunyong Choi, Moon-Ho Jo

**Affiliations:** 1 Center for Artificial Low Dimensional Electronic Systems, Institute for Basic Science (IBS), 77 Cheongam-Ro, Pohang 790-784, Korea; 2 Division of Advanced Materials Science, Pohang University of Science and Technology (POSTECH), 77 Cheongam-Ro, Pohang 790-784, Korea; 3 School of Electrical and Electronic Engineering, Yonsei University, Seoul 120-749, Korea; 4 Department of Chemistry and Division of Advanced Nuclear Engineering, Pohang University of Science and Technology (POSTECH), 77 Cheongam-Ro, Pohang 790-784, Korea; 5 Department of Materials Science and Engineering, Pohang University of Science and Technology (POSTECH), 77 Cheongam-Ro, Pohang 790-784, Korea

## Abstract

Two-dimensional stacks of dissimilar hexagonal monolayers exhibit unusual electronic, photonic and photovoltaic responses that arise from substantial interlayer excitations. Interband excitation phenomena in individual hexagonal monolayer occur in states at band edges (valleys) in the hexagonal momentum space; therefore, low-energy interlayer excitation in the hexagonal monolayer stacks can be directed by the two-dimensional rotational degree of each monolayer crystal. However, this rotation-dependent excitation is largely unknown, due to lack in control over the relative monolayer rotations, thereby leading to momentum-mismatched interlayer excitations. Here, we report that light absorption and emission in MoS_2_/WS_2_ monolayer stacks can be tunable from indirect- to direct-gap transitions in both spectral and dynamic characteristics, when the constituent monolayer crystals are coherently stacked without in-plane rotation misfit. Our study suggests that the interlayer rotational attributes determine tunable interlayer excitation as a new set of basis for investigating optical phenomena in a two-dimensional hexagonal monolayer system.

## Abstract (web summary)

Single atomic layers of transition metal dichalcogenides have outstanding electrical and optical properties. Here, the authors show that light absorption and emission from stacks of different transition metal dichalcogenide monolayers can be tuned by rotating the crystals.

## Graphical abstract

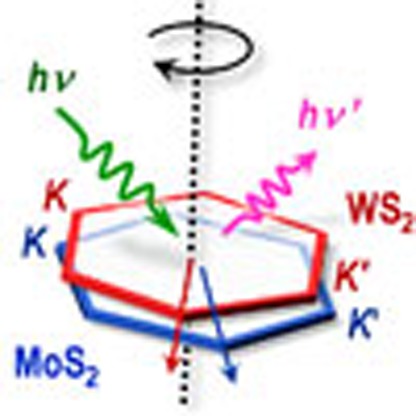

Hexagonal transition metal dichalcogenide monolayers (*h*-TMDCs MLs) form a new class of two-dimensional (2D) materials with extreme dimensional confinement effects^1^,^2^,^3^,^4^,^5^,^6^,^7^,^8^,^9^,^10^,^11^. In particular, large bandgap *h*-TMDC MLs such as MoS_2_, MoSe_2_, WS_2_ and WSe_2_ possess distinct singularities near the band edges, showing strong light–matter interactions in the visible range^1^,^12^,^13^,^14^,^15^,^16^. Artificial 2D superlattices, which are vertical stacks of dissimilar *h*-TMDCs MLs, are even more intriguing because upon light illumination, interlayer excitons can form across the constituent MLs at newly formed 2D electronic superstructures^17^,^18^,^19^; these excitons can recombine radiatively, thereby emitting light of different colours and intensities. This phenomenon strongly suggests that, upon inter-ML coupling, tunable light absorption and emission, as well as charge separation, can be achieved. Nevertheless, the relevant 2D band structure engineering has not been fully utilized^20^, mainly due to lack of control over the in-plane rotational misfit of hexagonal unit cells between the adjacent MLs^21^,^22^,^23^,^24^; this misfit may otherwise serve as an additional quantum index in defining the *h*-TMDC 2D superstructures^25^. For example, integration of discrete ML crystals into such 2D heterostructures usually entails mechanical exfoliation of individual MLs from bulk crystals, followed by manual transfer. By this method, the relative rotations of the individual hexagonal Brillouin zone in the stacks cannot be easily identified; consequently, the ‘interlayer' excitation cannot be immediately described^13^,^23^,^26^,^27^, although an example of a graphene/*h*-BN/graphene heterostructure has been described^28^. In *h*-TMDC ML stacks, the inter-ML bonding is inherently weak across van der Waals heterointerfaces, compared with the intra-ML bonding, so the ML stacking orientations of each hexagonal unit cell must sensitively determine the low-energy excitations.

In the following, we have investigated light absorption and emission at the heterointerfaces of ML stacks composed of MoS_2_ and WS_2_ MLs with and without inter-ML rotational misfit in both spectroscopic and time-resolved manners. We provide evidence that the interlayer excitation is intimately determined by the interlayer rotational degree of freedoms, which are tunable from direct- to indirect-gap transitions.

## Results

### Photocurrent spectra in MoS_2_/WS_2_ bilayer stacks

**Figure 1:**
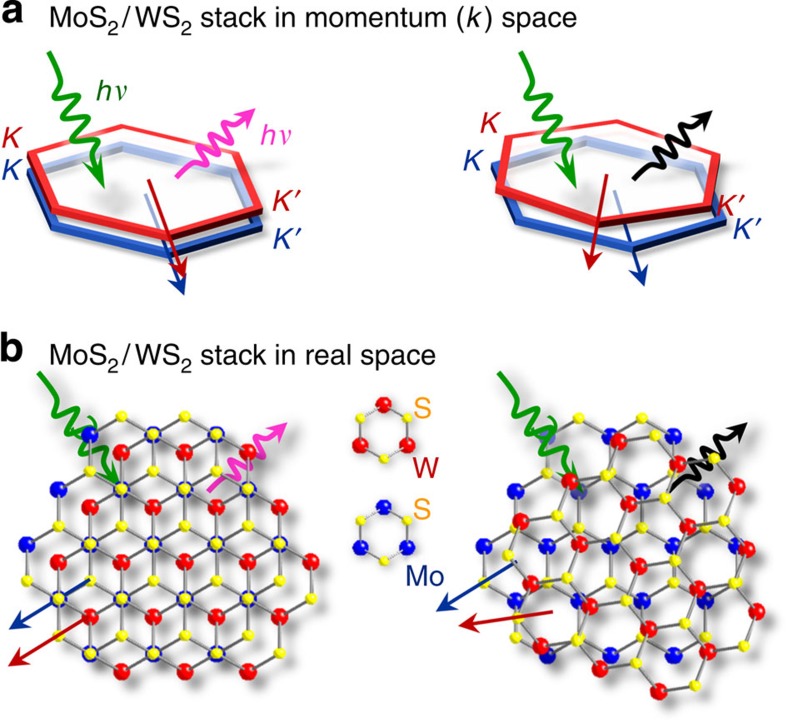
Schematics of this study for light absorption and emission by rotation of monolayer hexagonal semiconductors (MoS_2_ and WS_2_) in vertical stacks. (**a**) The vertical stack of MoS_2_ and WS_2_ in momentum (*k*) space. (**b**) The vertical stack of MoS_2_ and WS_2_ in real space.

**Figure 2:**
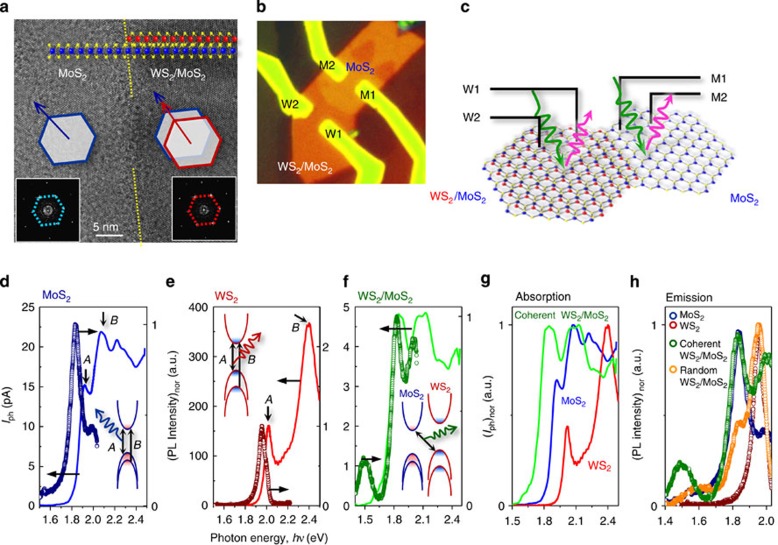
Rotational crystal structures of the MoS_2_/WS_2_ stacks and light absorption and emission characteristics. (**a**) In-plane transmission electron microscopy images of the vertical stacking. The lower insets: fast Fourier transform diffraction patterns at each region, showing the invariable hexagonal unit-cell orientations in the stack. (**b,c**) Optical microscope image of the MoS_2_/WS_2_ stack device and schematics of local light absorption and emission. (**d–f**) Photocurrent (*I*_ph_) and photoluminescence (PL) spectra of MoS_2_ (blue), WS_2_ (red) and coherently stacked MoS_2_/WS_2_ bilayer (green) by local light illumination. Comparison of (**g**) *I*_ph_ and (**h**) PL spectra.

We designed an experiment (Fig. 1a) that uses spatially, spectrally and time-resolved optoelectronic probes. We first prepared two different kinds of MoS_2_/WS_2_ stacks (Fig. 1b). Coherent bilayer stacks with hexagon-on-hexagon unit-cell stacking of MoS_2_ and WS_2_ MLs without incommensurate interlayer rotations were achieved by vertical heteroepitaxial growth. Both ML crystals have the same hexagonal crystal symmetry and are of similar size (MoS_2_: *a*=3.150 Å; WS_2_: *a*=3.153 Å). Therefore, the individual atom positions and thus the crystallographic ML stacking orders can be thermodynamically stabilized without rotational misfit (Fig. 1b). Random bilayer stacks were fabricated by manual stacking of individual MoS_2_ and WS_2_ MLs; thus the rotation-misfit order is unknown. Heteroepitaxial stacking growth, in which the second WS_2_ ML grows over the first MoS_2_ and WS_2_ MLs, occurred by edge nucleation (the top inset of Fig. 2a). Fast Fourier transformation of transmission electron microscope images (the bottom insets of Fig. 2a) confirmed that the in-plane orientations of the *h*-unit cells are identical; thus the bilayer stack is rotationally coherent across the clean van der Waals interface. To investigate light absorption and emission arising from both intra- and inter-ML transitions, we fabricated bilayer devices from partially covered MoS_2_/WS_2_ stacks (Fig. 2b,c), in which multiple electrical contacts were made in such a way that we can collect photoexcited carriers (photocurrent, *I*_ph_) spectrally by scanning with a focused supercontinuum laser. Our diffraction-limited laser beam spot is small enough to spatially resolve each ML within the bilayer stacks^29^,^30^,^31^. Due to the small absorption volume of ML crystals, direct probing of optical absorption of individual MLs without ensemble averaging is technically difficult; instead, we use local light illumination to collect the *I*_ph_ spectra, because this method can spectrally capture the local optical absorption and the subsequent photocarrier generation and transport. In the sense of the spectral responses, this measurement can be equivalent to optical absorption to a certain extent. For example, by locally illuminating only the bottom MoS_2_ ML, the collected *I*_ph_ with the M1–M2 electrode pair can represent the MoS_2_ intralayer absorption. Such *I*_ph_ spectra of MoS_2_ and WS_2_ MLs (Fig. 2d,e) are closely reminiscent of the equivalent absorption spectra, in that the spectral *I*_ph_ onset and peak positions represent the absorption edges and excitonic transitions, so-called *A* and *B* excitons, respectively. By illuminating the MoS_2_/WS_2_ stack regions, the *I*_ph_ arising from interlayer transitions can be selectively collected with the M1–W1 electrodes pair (Fig. 2f). This inter-ML *I*_ph_ spectral onset shifts down to 1.5–1.6 eV, far below the fundamental energy gaps (*E*_g_) of MoS_2_ MLs (*E*_g_=1.87 eV) and WS_2_ MLs (*E*_g_=1.96 eV) (Fig. 2g). Earlier theoretical calculations predict that the WS_2_/MoS_2_ junction forms a type-II band alignment with 1.58–1.60 eV interlayer *E*_g_ between the MoS_2_ valence band maxima (VBM) and the WS_2_ conduction band minima (CBM) (the inset of Fig. 2f)^12^,^17^,^18^,^19^; thus our observations of the photocurrent onset at 1.5–1.6 eV can be provisionally assigned to such an interlayer transition.

### Photoluminescence spectra in MoS_2_/WS_2_ bilayer stacks

Local light emission was investigated by measuring the photoluminescence (PL) spectra (Fig. 2d–f,h). Individual MLs of both MoS_2_ and WS_2_ typically exhibit a Stoke shift of ∼0.1 eV near the fundamental absorption edges, which indicates efficient radiative recombination from direct-bandgap transitions^14^. In addition, we observed substantial emission in the stack region at 1.5 eV with a Stoke shift >0.1 eV, which suggests the existence of another radiative recombination from inter-ML states. It is accompanied by a slight PL reduction at 1.8 eV, compared with the spectrum of individual MoS_2_ MLs. Provided that the oscillator-strength sum is conserved, this reduction can be understood to be due to split photoexcitation from the MoS_2_ VBM into the WS_2_ CBM and the MoS_2_ CBM. Another contribution from ultrafast charge separation into each ML before inter-ML recombination^32^ may also affect the relative spectral weight. By contrast, this low-energy emission is absent from the random stack (Fig. 2h), whereas it shows PL peaks from both MoS_2_ and WS_2_ at the higher excitation energy with pronounced PL suppression at 1.8 eV. This absence of the radiative recombination at 1.5 eV in the random stacks, together with their lower-energy photocurrent onset at 1.6–1.7 eV, confirms that the emission responsible is generated by the interlayer transition, which is effectively suppressed due to non-radiative recombinations by momentum mismatch or interfacial impurity centres in the random stacks.

### Interlayer transition dynamics in MoS_2_/WS_2_ bilayer stacks

**Figure 3:**
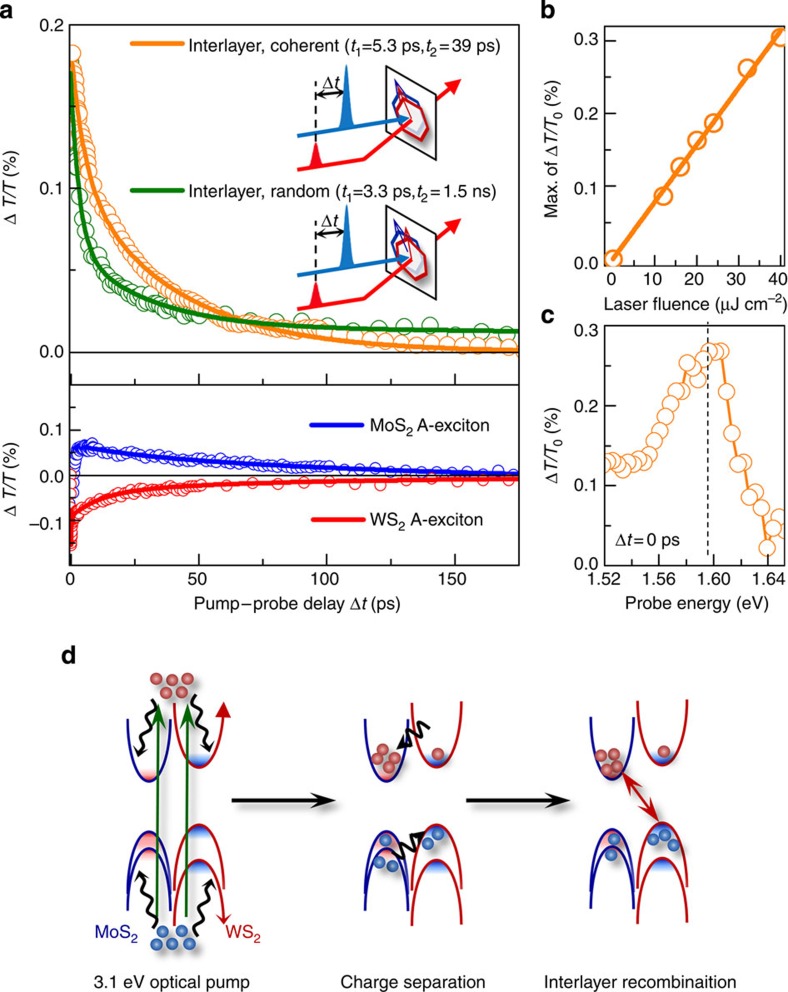
Ultrafast time-resolved exciton dynamics of coherently/randomly stacked bilayers. (**a**) Top panel: comparison of transient dynamics of interlayer excitons in coherently stacked and randomly stacked bilayers. Both bilayers are pumped by the same laser fluence of 24 μJ cm^−2^, and measured at the same probe photon energy of 1.6 eV. Bottom panel: intralayer exciton dynamics of MoS_2_ (blue, 1.87 eV) and WS_2_ (red, 1.96 eV) measured in the coherently stacked bilayer with the same pump fluence of 24 μJ cm^−2^. (**b**) The pump-fluence-dependent maximum Δ*T*/*T*_0_ of the coherently stacked bilayer. (**c**) Spectrally resolved Δ*T*/*T*_0_ at the direct interlayer exciton state of ∼1.6 eV (the dashed line) is shown. The data are measured at Δ*t*≈0 ps with a pump fluence of 32 μJ cm^−2^. (**d**) Schematic illustration of a pump-induced exciton transient as a function of pump–probe delay Δ*t* (from left to right). Immediately after the 3.1-eV pump excitation (left), electrons are rapidly transferred in MoS_2_ and holes are rapidly transferred in WS_2_ (middle). Then the interlayer recombination is measured by setting the probe photon energy of 1.6 eV (right).

To further investigate the observed contrast in interlayer transitions between the coherent and random stacks, we used ultrafast time-resolved optical pump–probe spectroscopy to study the inter-ML transition dynamics. The stacks were excited using a 50-fs, 3.1-eV pump pulse, and the corresponding differential transmission data (Δ*T*/*T*_0_, where Δ*T* is the pump-induced transmission change, and *T*_0_ is the transmission without the pump) from 1.47 to 2.13 eV probe photon energy were measured as a function of pump–probe delay Δ*t*^33^ (the inset of Fig. 3a; see also Supplementary Fig. 1). The two kinds of stacks were excited by the laser pump at a fluence of 24 μJ cm^−2^. Immediately after the pump, the signals for the interlayer exciton with 1.6 eV probe (both of coherent and random stacks) rapidly increase with Δ*T*/*T*_0_>0 (the upper panel of Fig. 3a). The positive Δ*T*/*T*_0_ originates from the reduced probe absorption by state-filling in the interlayer exciton states, that is, the Pauli-blocking effect. The kinetic origin of the increased interlayer transients is rapid charge separation into each ML; the photoexcited electrons and holes are immediately transferred into energetically favoured states in MoS_2_ and WS_2_, respectively^33^, as illustrated in the left and middle panels of Fig. 3d. This rapid charge transfer and resulting interlayer exciton population explain the measured PL intensity ratio (Fig. 2f,h); if the charge transfer is slow compared with the intralayer exciton lifetime of individual MLs, no interlayer PL can be expected. It also signifies that in the bilayer stacks, the charge-transfer dynamics determines the relative PL intensities between the inter- and intra-ML light emissions. In fact, the linear fluence dependence of the peak Δ*T*/*T*_0_ in Fig. 3b ensures that no higher-order nonlinear excitonic interactions are involved; that is, that first-order population dynamics primarily governs both the PL and the Δ*T*/*T*_0_ dynamics^34^. The spectrally resolved prominent Δ*T*/*T*_0_ peak at 1.6 eV measured at *Δt*≈0 ps (Fig. 3c) corroborates that the rapid charge separation leads to the interlayer population dynamics, rationalizing the interlayer excitonic PL in the coherent bilayer stacks. Although both stacks exhibit rapid charge separation, the corresponding decay dynamics show qualitatively different features, as indicated in bi-exponential function fits with fast (*τ*_1_) and slow (*τ*_2_) decay components. The initial decay was slower in the coherently stacked bilayer (*τ*_1_=5.3 ps) than in the randomly stacked bilayer (*τ*_1_=3.3 ps). This difference indicates that coherent stacks were less sensitive to defect-mediated recombination than were the random stacks, presumably because the coherent stacks have cleaner interfaces with fewer defects than the random stacks^35^. More importantly, for the slow decay component, *τ*_2_ was much faster in the coherent stacks (*τ*_2_=39 ps) than in the random ones (*τ*_2_=1.5 ns). This ultrafast timescale is comparable to direct-gap transitions in conventional III–V semiconductor superlattices^36^, and is qualitatively different from previous reports of random bilayer stacks of WSe_2_/MoS(Se)_2_ MLs, in which *τ*_2_ ranges from nanoseconds to microseconds, attributed to indirect-gap interlayer recombinations^4^,^6^. The substantially faster interlayer recombination dynamics of ∼39 ps in our coherent stacks is comparable to those typically observed in the intralayer direct-gap transitions in individual MoS_2_ MLs^37^,^38^. This similarity strongly suggests that our interlayer transitions may be like those of direct gaps. The slower transient character (nanoseconds) in the random stacks is consistent with the observed absence of the corresponding PL at 1.5 eV. This fast recombination dynamics of *τ*_2_ excludes a possibility that the excitons are recombined radiatively from defect states, by which the corresponding decay timescale would be very long in the range of nanoseconds^39^. Overall, the observed contrasts in interlayer transitions of the coherent and random stacks for both spectral and dynamic features suggest critical roles of the relative rotation misfit in the 2D hexagonal *k*-space of the bilayer stacks.

### Interlayer band structure calculation

**Figure 4:**
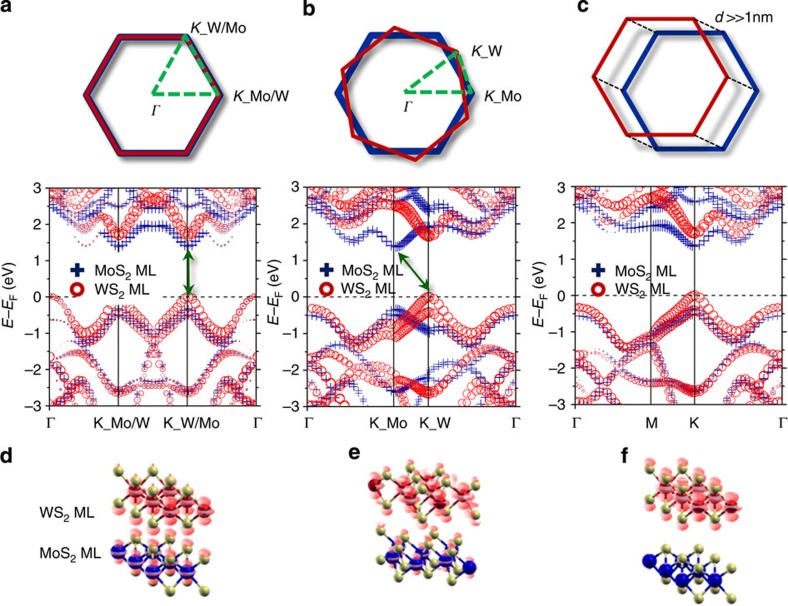
Band structures of coherent and random stacking MoS_2_/WS_2_ bilayers and partial charge density plot of MoS_2_/WS_2_ bilayers. Band structures of (**a**) coherent and (**b**) random stacks. Here we consider random stacking with a rotation by 21.79° between two MLs. The upper figures represent the overlap view of the primitive cell Brillouin zones for each stacking type. Dashed lines on the Brillouin zone indicate the **k** path, which the band structures are calculated along. The coherent and random stacking bilayers show the direct- and indirect-bandgap features, respectively. Each contribution from MoS_2_ ML and WS_2_ ML is denoted by the size of the blue crosses and red circles, respectively. (**c**) The band structure of coherent stacking with large distance (*d*≫1 nm) between two layers. (**d**–**f**) The partial charge density plot of the structure used in **a**–**c** at the *Γ* point in the VB.

We performed band structure calculation of MoS_2_/WS_2_ bilayer for both coherent and random stacks (Fig. 4), in which two Brillouin zones of each ML are stacked atop each other. In principle, one must consider random displacements and rotations of the two hexagonal unit cells for the random stacks. However, we only considered the rotational symmetry (Fig. 4a,b; Supplementary Fig. 2), because the crystal momentum, and thus the consequent band dispersion is invariant and has a simple displacement between two MLs. The coherent stacking (Fig. 4a), shows a direct-bandgap feature at the *K*-point. At the *K*-point, the VBM mainly come from the WS_2_ ML and spin–orbit (SO) splitting of 0.42 eV is evident. The VB of the MoS_2_ ML, which has smaller SO splitting of 0.15 eV at the *K*-point, is well below the VBM. Thus, one can expect that the valley polarization in VB is induced mainly by the WS_2_ ML. At the CBM, the orbital character mainly comes from the MoS_2_ ML, and the SO splitting is almost negligible. Meanwhile, the random stacks show indirect-bandgap features, as exemplified for the 21.79°-rotated bilayer (Fig. 4b). The difference in the recombination time in our study may be due to this change in direct- and indirect-gap interlayer transitions. The random stacks also show a clear difference in the inter-ML orbital interactions from those of the coherent stacks. In the coherent stacks, the VBM is almost degenerate at the *K* and *Γ* points; notably, strong interlayer orbital mixing occurs at the *Γ* point, at which the main orbital contribution comes from S *p*_*z*_ orbitals of both MoS_2_ and WS_2_ MLs. Thus, instead of the distinct direct-bandgap structure in each MoS_2_ or WS_2_ ML (Supplementary Fig. 3), the MoS_2_/WS_2_ bilayer displays both direct- and indirect-gap features. In the extremely strong interlayer interaction limit (effectively as in bulk crystals), this system shows clear indirect-bandgap features between *K* at CBM and *Γ* at VBM^40^. However, in the random stacks the *Γ* point is downshifted compared with the *K*-point, and the orbital mixing becomes much weaker; due to the rotational misfit in the random stacking, the orbital overlap between S *p*_*z*_ orbitals from each ML becomes much smaller, and the VBM at the *Γ* point is mainly contributed from the WS_2_ ML. Such interlayer orbital interaction can be identified from the charge density plot of the orbitals at the *Γ* point (Fig. 4d,e). We attempted to correlate the interlayer interaction with the band structure and the associated charge recombination. Figure 4c shows the band structures of the well-separated MoS_2_/WS_2_ bilayer (thus inducing effectively small interlayer orbital interaction (Supplementary Fig. 3 for the inter-ML distance dependence) without rotational misfits. The *Γ* point energy of the VB decreases as the interlayer distance increases, forming the direct bandgap at the *K*-point (Supplementary Fig. 3). At the same time, the orbital of the VBM at the *Γ* point originates mostly from the WS_2_, with the interlayer orbital mixing being much smaller. The charge density plot (Fig. 4f) supports our hypothesis that interlayer orbital mixing was suppressed, as observed for the random stack. With strong interlayer interaction, recombination centres can form at the *Γ* point, thereby reducing the exciton lifetime, as observed in our experiments^41^. Because the photogenerated holes are distributed to the *Γ* point with the mixed orbital characters of both MLs, the recombination occurs in similar timescales in the inter-ML recombination. Nevertheless, if the interlayer interaction diminishes due to the interlayer distance or the rotation misfit, the orbital mixing between two MLs weakens and recombination lifetime lengthens, possibly with inelastic scattering processes. We find qualitatively the same conclusion from a calculated optical absorption of the MoS_2_/WS_2_ stack using the Bethe–Salpeter equation method, in which the absorption spectrum of the stack is significantly different from the sum of the spectra of the two MLs with a large absorption cross-section below the *E*_g_ of MoS_2_ and WS_2_ MLs, due to the interlayer transition^12^.

## Discussion

We tested the hypothesis of interlayer charge separation and direct-gap recombination by further examining the timescale of individual *A*-exciton transients of MoS_2_ ML (1.87-eV probe) and WS_2_ ML (1.96-eV probe) as in the lower panel of Fig. 3a. Near Δ*t*≈0, we observed photoinduced bleaching^42^ and absorption of the *A* excitons in MoS_2_ and WS_2_, respectively, that is, Δ*T*/*T*_0_>0 for MoS_2_ and Δ*T*/*T*_0_<0 for WS_2_ (refs 33, 43, 44). All Δ*T*/*T*_0_ signals decayed in ∼100 ps, except in the random stack, in which the intralayer *A* excitons of MoS_2_ ML and WS_2_ ML are well known to be direct-gap transitions. Thus, the faster decay of the Δ*T*/*T*_0_ in the coherent stacks than in the random stacks strongly supports the direct-gap recombination, in direct comparison with the indirect-gap recombination in the random stack, as they are also consistent with our model calculation. Our observations suggest that the *h*-TMDC ML stacking orientation is an additional quantum attribute in inter-ML exciton phenomena, and also demonstrate the achievability of tunable interlayer optical excitations for novel 2D photonics and photovoltaics based on *h*-TMDC MLs.

## Methods

### Spatially and spectrally resolved photocurrent spectroscopy

For the spectral photocurrent measurements, we used a broadband supercontinuum laser (450≤*λ*≤2,000 nm) combined with a monochromator to obtain high-resolution spectra. During the wavelength scanning, photocurrent was measured using a lock-in technique with a chopper frequency of 500 Hz and then normalized to the photon flux. The chopped laser beam was focused by a microscopic lens (numerical aperture=0.8) and illuminated the ML devices.

### MoS_2_/WS_2_ bilayer band calculations

The band structure calculations were performed using the Vienna *ab initio* package (VASP)^45^. The projector-augumented wave method with the PBE-sol exchange-correlation functional^46^ was used for the density functional theory calculation. The plane-wave cutoff was set to 500 eV and a 10 × 10 × 2 *k*-mesh was used for the self-consistent calculation. SO coupling was considered in this calculation. To consider the bilayer effect, calculations assumed a vacuum thickness of ∼10 Å.

## Additional information

**How to cite this article:** Heo, H. *et al.* Interlayer orientation-dependent light absorption and emission in monolayer semiconductor stacks. *Nat. Commun.* 6:7372 doi: 10.1038/ncomms8372 (2015).

## Supplementary Material

Supplementary Information Supplementary Figures 1-3 and Supplementary References
