# Supplementary material for: Interlayer orientation-dependent light absorption and emission in monolayer semiconductor stacks
Source: Nat Commun. 2015 Jun 23;6:7372. doi: 10.1038/ncomms8372 (PMC4557351; doi:10.1038/ncomms8372)
Supplement: Supplementary Information — Supplementary Figures 1-3 and Supplementary References [file ncomms8372-s1.pdf]

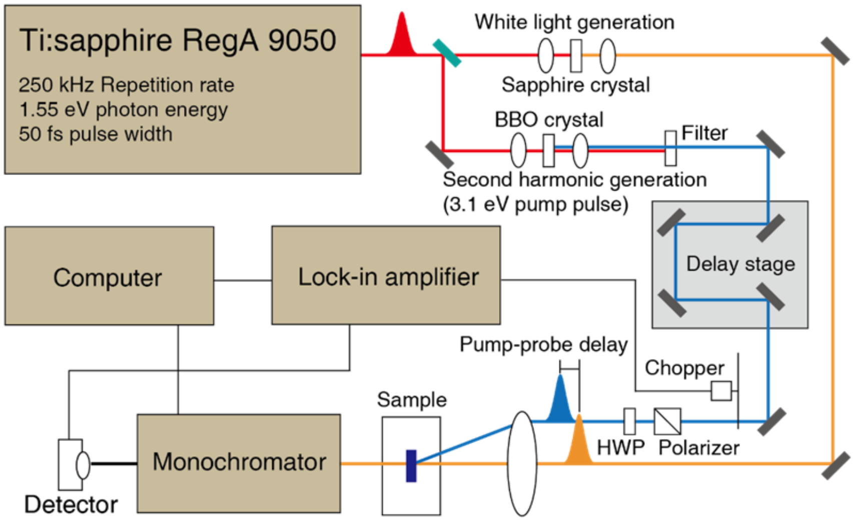

Supplementary Figure 1. Ultrafast time-resolved optical pump-probe spectroscopy. Ultrafast visible-pump and white-light probe spectroscopy was performed in coherently and randomly stacked bilayers. The experimental setup is shown in Figure SX. The laser system is based on the 250 kHz ultrafast Ti:sapphire regenerative amplifier (Coherent RegA 9050). A portion (70 %) of RegA output (1.55 eV) is frequency-doubled in a 1 mm BBO crystal to generate the 3.1 eV pump pulse. Remaining 30 % is focused on a 1 mm-thick ultra-clean sapphire plate to generate the white-light supercontinuum. The group-velocity dispersion is corrected by following the method described in the previous report.[1] An objective lens (Mitutoyo M Plan Apo ×10) is used to focus both pump and probe pulses on the MoS_2-WS_2 heterostructure sample, where the pump and probe spot size (FWHM) are measured to be 20 μm and 10 μm, respectively. Because of the dispersive optics in the objective lens, the cross-correlation of the white light and the 3.1 eV pulse is large, resulting in ~ 300 fs duration, which limits the minimum time-resolution in our experiment.[2] The polarizations of the pump and probe beams are linearly orthogonal with each other to prevent coherent artifacts near zero time delay. The transmitted probe pulse through the sample is collected and directed into a monochromator

(Oriel Cornerstone 260 1/4 m), and the ΔT signal is measured by a Si photodetector. High sensitivity for $\Delta T/T_0 \approx 10^{-5}$ was achieved with the mechanical chopping of the pump pulse at a frequency of 10 kHz, which also reduced the $1/f$ noise. The pump-induced transmission ΔT is measured by the phase-sensitive lock-in amplifier (SR850). The sign of ΔT is checked either by the so called "two diode method"[3] or by the thin-film Bi epilayer sample. All measurements were performed at room temperature with the samples equipped in a vacuum microcryostat (Janis Model ST-300MS).

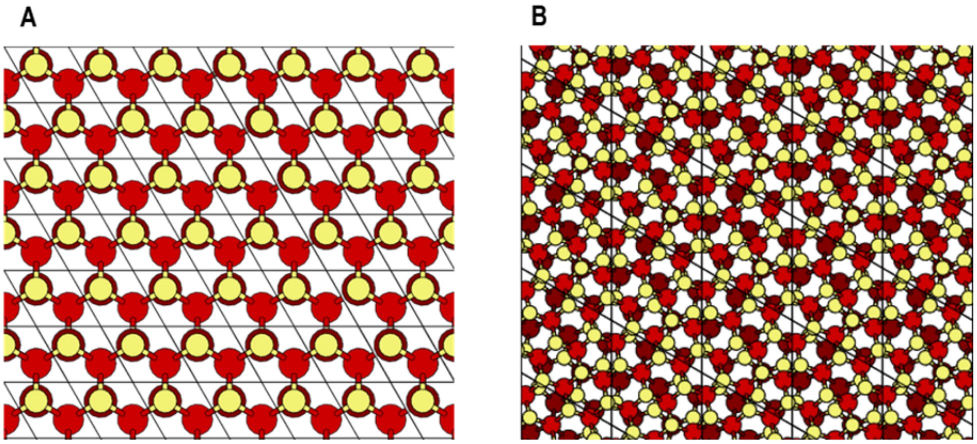

Supplementary Figure 2. **The in-plane crystal structure of coherent and random MoS_2/WS_2 bilayer stacks for our calculations.** (**a**) The coherent stacking is from the usual 2H-MX_2 stacking type (**b**) the random staking is formed with a rotational misfit of 21.79°. There are seven formula units of MoS_2 and WS_2 in the unit cell of random stacking. Mo, W, and S atom are denoted by brown, red, and yellow colors, respectively. The solid line indicates the unit cell of coherent and random stacking.

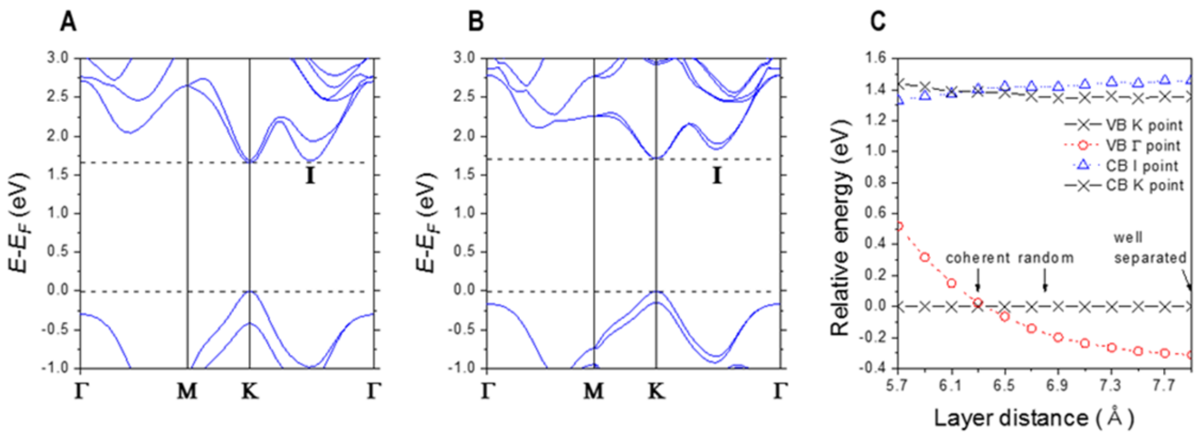

Supplementary Figure 3. **Band structures of WS_2 and MoS_2 MLs and the energy change of band edges as the function of interlayer distance.** (**a,b**) The VBs of WS_2 and MoS_2 MLs do not show degeneracy at the Γ and K points, while the MoS_2/WS_2 bilayer show degeneracy in the VBM. The band structures of elongated MoS_2/WS_2 bilayer in Figure 4c are effectively the same as the overlap of these two band structures with the type-II band alignment. (**c**) The energy change of MoS_2 band edge as a function of interlayer distance. The energies of the K and Γ points of the VB and K and I point of the CB are referenced to the energy of the K point in the VB. The interlayer distance is defined by the distance between the W layer and the Mo layer. The energy of the Γ points in the VB sensitively decreases as the interlayer distance increases, and the energy of I point is rather insensitive to the interlayer distance. With the larger distance, it shows a direct band gap feature at the K point. The distances for the coherent bilayer, random stacking bilayer, and well-separated bilayer shown in Figure C are indicated with the arrows.

Supplementary Reference

1. Sim, S. *et al*. Exciton dynamics in atomically thin MoS_2: interexcitonic interaction and broadening kinetics. *Phys. Rev. B*. **88**, 075434 (2013).

2. Wang. Q. *et al*. Valley carrier dynamics in monolayer molybdenum disulfide from helicity-resolved ultrafast pump-probe spectroscopy. *ACS Nano*. **7**, 11087-11093 (2013)

3. Kim, J. *et al*. Ultrafast zero balance of the oscillator-strength sum rule in graphene. *Sci. Rep.* **3**, 2663 (2013).
